# Assessing the core functions of primary care in the Abomey-Calavi – So-Ava health district in Benin: A cross-sectional study

**DOI:** 10.4102/phcfm.v18i1.5547

**Published:** 2026-10-27

**Authors:** Rémi Danhoundo, Kéfilath Bello, Elias Martinien Avahoundje, Sorel Lokossou, Jean-Paul Dossou

**Affiliations:** 1 Center for Research on Human Reproduction and Demography (Cerrhud), Cotonou, Benin

**Keywords:** primary care, healthcare quality assessment, PCAT, health system performance, patient-centred care, Benin

## Abstract

**Background:**

Primary care quality in Benin was assessed using various tools, but without a focus on the core functions.

**Aim:**

To assess the core functions of primary care in the health district Abomey-Calavi – So-Ava.

**Setting:**

The study was conducted in primary care facilities in the Abomey-Calavi – So-Ava health district, Benin.

**Methods:**

This was a descriptive cross-sectional survey that used the validated primary care assessment tool (PCAT) for sub-Saharan Africa. Data were collected from 399 randomly selected patients across 27 health facilities using the PCAT via the REDCap mobile application. Univariate and bivariate statistical analyses were conducted using R software.

**Results:**

Affiliation to the facilities was high among 47.1% of users. Users globally rated the health district’s primary care performance as average. Health system coordination was scored as poor. First-contact access, first-contact utilisation, comprehensiveness, continuity of care, and coordination – information system were scored as average. Only the person-centred care domain was scored as good. Those who rated their health as good or excellent tended to give higher scores. Also, users of private facilities reported slightly higher score than users of public facilities.

**Conclusion:**

The performance of primary care was globally scored as average. This PCAT looks useful for assessing the core functions of primary care and provided novel information on performance.

**Contribution:**

This study demonstrates how the sub-Saharan African version of the PCAT can be used to generate context-specific evidence on the performance of core primary care functions at district level.

## Introduction

The Millennium Development Goals era witnessed significant improvements in global health outcomes. However, substantial gaps remain in individuals’ ability to attain the highest possible standard of health.^1^ Approximately half of the global population lacks access to core healthcare services, and ill-health disproportionately affects individuals exposed to unfavourable social and other determinants of health, resulting in persistent health inequalities within and across countries.^2^ In response to these challenges, primary health care (PHC) has been internationally recognised as a cornerstone of health system strengthening, given its proven links with improved health outcomes, equity, health security, and cost-effectiveness.^1^ Improving PHC has therefore been a global priority since the Alma-Ata Declaration in 1978,^3^ reinforced by the Astana Declaration in 2018,^4^ which identified three components for an effective implementation of PHC: (1) integrated health services, (2) multisectoral policies and actions addressing the determinants of health, and (3) empowerment and participation of individuals and communities. Within the first component, primary care, defined as first-contact care intended to address the large majority of population health needs, plays a central role in the success of PHC and in improving population health outcomes.^5,6,7^ According to the World Health Organization (WHO), effective primary care must fulfil five essential functions: First-contact accessibility, continuity, comprehensiveness, coordination, and people-centredness.^5,6,8,9^ Regular assessment of these functions is critical to ensuring quality, identifying gaps, guiding improvement efforts, and monitoring progress over time. Despite substantial investments in PHC, limited attention has been paid to the systematic measurement of primary care performance.^10^ Although several instruments are routinely used in sub-Saharan Africa to assess health system performance and service quality, none comprehensively capture these five core functions. For example, the Service Availability and Readiness Assessment (SARA), widely implemented across African countries,^11^ provides valuable information on infrastructure, staffing, and service availability, but offers limited insight into care processes and the quality of services delivered, including primary care. The global Primary Health Care Performance Initiative (PHCPI) attempted to assess the core functions of primary care^12^ through collaboration with selected sub-Saharan African countries using routine data. However, in low- and middle-income settings, the lack of ‘big data’ and detailed information on care processes constrains routine measurement of these functions. Under PHCPI, for instance, person-centred care was captured by a single indicator (the percentage of parents or caregivers informed of their sick child’s diagnosis), continuity was approximated using the diphtheria, tetanus, pertussis 3-dose series (DTP3) dropout rate, and coordination was not included in the quality score. In its PHC measurement framework, WHO therefore recommends assessing core primary care functions using patient-reported experiences collected through facility exit interviews or population-based surveys.^8^ The primary care assessment tool (PCAT) has been adapted and applied in several sub-Saharan African countries, including South Africa, Malawi, Kenya, and Uganda.^13,14,15,16,17,18^ Building on this experience, a collaborative effort recently produced a regional version of the PCAT for sub-Saharan Africa, adapted to contextual realities and designed to enable standardised measurement of primary care functions across African countries.^19^ Pilot studies were subsequently planned to test this tool in real-world settings, including in Benin.

Benin was among the first African countries to adopt PHC, embedding it in public health policies since the Alma-Ata conference and reinforcing it through the Bamako Initiative (1987) and the Astana declaration.^20,21^ National efforts have focused on strengthening service delivery and community health, including the adoption of a national community health policy in 2019.^22^ However, like many African countries, Benin still lacks routine data to assess and track primary care performance. Existing evaluations mainly focus on service availability and readiness (such as SARA)^23,24^ and health outcomes (for example, vaccination coverage and service utilisation reported in annual health statistics), leaving the ‘black box’ of care processes largely unexplored and limiting their usefulness for quality improvement. The Manual for the Assessment and Monitoring of Patient Safety in First-Contact Health Facilities in Benin, published by the Benin Health Sector Regulation Authority in 2024, represents a positive step by introducing standards related to patient participation in decision-making (Domain B) and service coordination (Domain C). However, neither this manual nor other existing tools comprehensively capture all core primary care functions.^25^ Against this backdrop, the PCAT offers a unique opportunity to strengthen primary care assessment in Benin and support government efforts to improve quality, equity, and patient safety. This study applies the regional PCAT in the Beninese context, specifically in the Abomey-Calavi – So-Ava health district. Its objective is to evaluate the core functions of primary care, thereby providing actionable evidence for local quality improvement and contributing to strengthening routine performance monitoring aligned with PHC principles.

## Research methods and design

### Study design

This was a descriptive cross-sectional survey conducted in the Abomey-Calavi – So-Ava health district in Benin.

### Study setting

Benin is a francophone West African country. The health system in Benin is organised into 34 health districts. The health district is a local health system which includes all public and private primary care facilities, the district hospital and a network of community health workers. Health activities in a health district in Benin are coordinated by a health district management team led by a district medical officer.^26^

This study, focusing on primary care facilities, was conducted in the Abomey-Calavi – So-Ava health district. This health district is in southern Benin. It was purposively selected in consultation with the Ministry of Health (MOH), based on the following criteria:

- the possibility of having a wide variety of contexts within the health district (public or private, urban or rural, etc.)
- the need expressed by the district medical officer to measure the quality of care in the health district
- the geographical accessibility of the health district due to budget constraints.

The health district covers two communes (Abomey-Calavi and So-Ava), with a population of around 972 735 in 2021. It has 206 healthcare facilities, comprising 25 public, 167 private liberal and 14 private religious or associative facilities. The curative health service utilisation rate was 43.5%, and maternal mortality was 149.8 per 100 000 live births in the health district in 2021.^27^

### Study population and inclusion criteria

The study population included all primary care users in the Abomey-Calavi – So-Ava health district. To be included, users needed to be aged 18 years or older (those accompanying patients under 18 years were interviewed, providing indirect information on the care of minors) and to have visited the healthcare facility at least three times in the last 2 years (to ensure sufficient experience with the facility). Users who were unable to answer the questions due to a serious mental disorder or severe acute illness were excluded.

### Sample size

We determined the sample size using Schwartz’s formula. Key assumptions were a 95% confidence level, a 5% margin of error, and 50% scoring three or more in any domain (which was considered the minimum acceptable score). This formula gave a sample size of 384. This sample size was increased by 5.5% (*n* = 405) to account for any withdrawals from the study.

### Sampling method

A proportional stratified multistage sampling approach was employed to select users for inclusion in the study.^28^ A list of health facilities within the health district was obtained from the MOH. Based on geographical location, the facilities were first stratified into two primary strata: Abomey-Calavi commune and So-Ava commune. The sample allocated to each stratum was proportional to the number of deliveries recorded in the respective commune, with the number of deliveries serving as a proxy for attendance. Indeed, among the services for which data were available, deliveries were the most frequently used. Each stratum was further subdivided into sub-strata according to the type of health facility (public or private). Within each sub-stratum, the sample allocation also followed the proportion of deliveries. The number of facilities to be selected in each sub-stratum was calculated by dividing the expected number of participants by 15, assuming an average of 15 eligible users per facility. In So-Ava commune, the only private facility was included by default. For the remaining sub-strata, facilities were selected through simple random sampling.

Figure 1 illustrates the sampling procedure, including the sample proportions for each sub-stratum and the number of facilities included in each.

**FIGURE 1:**
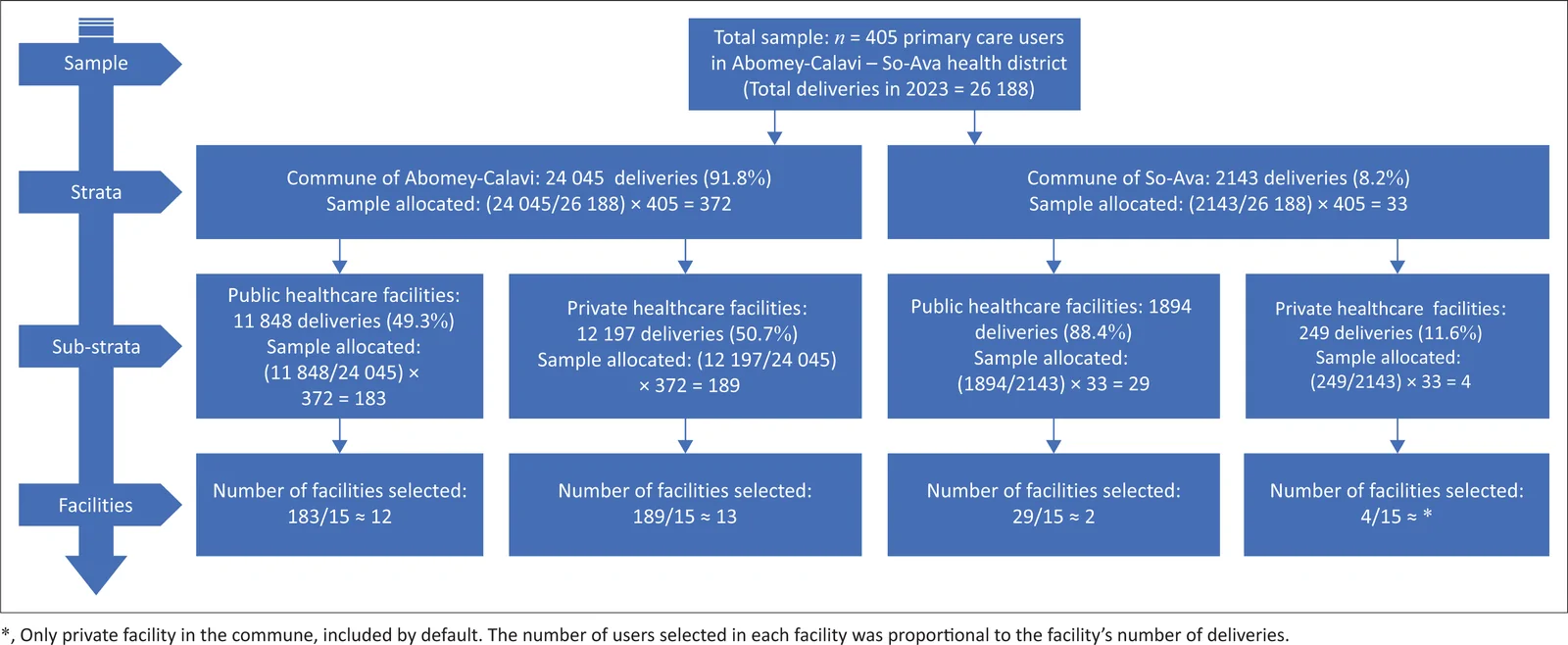
Illustration of the sampling procedure.

A discussion was held with the selected facility managers to assess the feasibility of data collection in their facility and obtain their consent. At each healthcare facility, research assistants interviewed every second user who had used care during the data collection period and met the inclusion criteria. Users were invited to participate upon leaving care. If a user refused, the next user was selected.

### Data collection

The sub-Saharan Africa version of the Primary Care Assessment Tool (PCAT-SSA) was used.^19^ Each core function of primary care (corresponding to one PCAT domain; Table 1) was measured by several items, rated on a Likert scale from ‘certainly not’ (1) to ‘certainly’ (4). The tool also provides items that measure affiliation with the primary care facility. Health status was self-reported by participants via questions that asked them to state their health status and whether they had a chronic health problem. Socio-demographic variables were declared by the participants.

**TABLE 1:** Definitions of the primary care assessment tool domains.

Domain	Definition
First contact (utilisation)	Utilisation of primary care services when a need for care arises. First contact refers to the primary care services being responsible for assisting the person in need of care to enter the healthcare system for each non-referred provision of healthcare.
First contact (access)	Care is first sought from accessible primary care services when a new health or medical problem arises. Primary care serves as the usual entry into the healthcare system.
Continuity	Longitudinal use of a regular source of care over time regardless of the presence or absence of disease or injury. A healthcare home is then established in which the person seeks continuous care, builds a long-term relationship with the provider, and fosters mutual understanding of expectations and needs.
Coordination (information system)	Availability of mechanisms to communicate information and use of that information in the person’s care plan.
Coordination (health system)	Linking of healthcare visits and services so that patients receive care for all their physical and health problems. Primary care systems taking responsibility and obligation to transfer information to and receive it from other sources involved in the patient’s care. This sub-function is assessed only when patients are referred to other providers.
Comprehensiveness (services available)	Availability of a wide range of core health services in primary care that promote and preserve people’s health as well as providing care for illness and disability.
Person-centredness	Provision of services that focus on what matters to the people served.

*Source:* Adapted from Bresick G, Von Pressentin KB, Mash R. Evaluating the performance of South African primary care: A cross-sectional descriptive survey. S Afr Fam Pract. 2019; 61(3):109–116. https://doi.org/10.1080/20786190.2019.1596666

The PCAT questionnaire was translated from English into French. This translation followed the rules for translating scientific documents as recommended by the WHO.^29^ The questionnaire was thus translated from English into French (translation) and then from French back into English (back-translation) to ensure that the French version was accurate and understandable to the Beninese population. Research assistants were trained to administer the questionnaire. During training, one and a half days were dedicated to oral translation into Fongbé and repeated practice sessions. The questionnaire was piloted with local users in French and Fongbé prior to data collection. The French version of the questionnaire was made available electronically via the REDCap application (Vanderbilt University, Nashville, Tennessee, United States) on tablets made available to research assistants. The questionnaire was mostly administered in French and, in a few cases, in Fongbé. The research assistants were two women and four men with experience in health data collection. At each facility, they first met with the facility manager, then identified a nearby location outside the facility to conduct the interviews. Every second user leaving the facility was approached; those who met the selection criteria and consented were interviewed. Ethical approval for this study was obtained prior to data collection. The study was conducted in accordance with the committee’s ethical requirements and approved protocol.

### Data analysis

Statistical analysis was carried out using R software (Version 4.5.1, R Core Team, R Foundation for Statistical Computing, Vienna, Austria). Missing data were assessed prior to analysis. Of the 399 questionnaires, 395 (99.0%) were complete and 4 (1.0%) contained at least one missing value. Given the very low proportion of missing data, no imputation procedure was performed. Analyses were conducted using available-case analysis, whereby participants with missing values were excluded only from analyses involving the affected variables.

Univariate analysis described socio-demographic characteristics, health status, and affiliation of the respondents. Socio-demographic data were presented as frequencies. Affiliation was calculated from responses to three questions designed to determine whether the patient attended another health institution and, if so, which institution knew him best. Responses were categorised and presented as frequencies. Using a univariate approach, we calculated a mean score for each core function from the responses (measured by a Likert scale from 1 to 4) to the sub-items of that function. An overall score was then calculated from the responses to all the sub-items. Scores were summarised using medians and interquartile ranges (IQRs), and categorised as very poor (< 1.5), poor (1.5–2.4), average (2.5–3.4) and good (≥ 3.5) performance.^30^ The proportion of respondents with a median score of 3 or higher in each domain was also computed, as this score was considered the minimum acceptable quality of primary care.^17^ A bivariate analysis examined associations between the primary care score and selected characteristics of individuals and facilities (socio-demographic variables, health status, institutional status, urbanisation status, sub-district – commune). For categorical selected characteristics, one-way analysis of variance (ANOVA) was used. The correlation between age and the primary care score was assessed using Spearman’s rank correlation. The significance level was set at *p* < 0.05.

### Ethical considerations

Ethical clearance to conduct this study was obtained from the *Comité d’Éthique de la Recherche de l’Institut des Sciences Biomédicales Appliquées* (CER-ISBA) (No. 199).

## Results

Data were collected from 399 users. Questionnaires collected from 395 (99.0%) users were complete. Missing data were minimal, affecting only four participants and a small number of socio-demographic variables. Users’ socio-demographic and health characteristics are presented in Table 2. The median age was 28 years (23.0–33.0), and 361 (90.5%) were women. The local language, Fongbé, was the native language of 165 respondents (41.4%).

**TABLE 2:** Socio-demographic and health characteristics.

Variable	*n*	%	Med	Q1–Q3
**Are you (gender) (*n* = 399)**	-	-	-	-
Men	38	9.5	-	-
Women	361	90.5	-	-
Other	0	-	-	-
**What is your age in years?**	-	-	28.0	23.0–33.0
**What is your home language? (*n* = 399)**	-	-	-	-
Fongbé	165	41.4	-	-
Adja	46	11.5	-	-
Aïzo	57	14.3	-	-
Toffin	36	9.0	-	-
Mina	22	5.5	-	-
Goun	19	4.8	-	-
Other	54	13.5	-	-
**Which of the following best describes your work situation now? (*n* = 397)**	-	-	-	-
Full-time employees	30	7.6	-	-
Part-time employees	13	3.3	-	-
Self-employed (informal sector)	144	36.3	-	-
Self-employed (formal sector)	130	32.7	-	-
Student	15	3.8	-	-
Homemaker	55	13.9	-	-
Retired or pensioner	3	0.8	-	-
Unemployed	7	1.8	-	-
**Which of the following best describes your schooling? (*n* = 398)**	-	-	-	-
Never went to school	79	19.8	-	-
Did not finish primary school	94	23.6	-	-
Completed primary school	14	3.5	-	-
Did not complete secondary school	130	32.7	-	-
Secondary school diploma	32	8.0	-	-
Higher education diploma, for example, bachelor’s degree, master’s degree	49	12.3	-	-
**Would you say your health status is? (*n* = 399)**	-	-	-	-
Excellent	76	19.0	-	-
Very good	122	30.6	-	-
Good	126	31.6	-	-
Fair	70	17.5	-	-
Poor	5	1.3	-	-
**Do you have a chronic condition? (*n* = 399)**	-	-	-	-
Yes	52	13.0	-	-
No	347	87.0	-	-
**Do you have piped water inside your house? (*n* = 398)**	-	-	-	-
Yes, inside house	117	29.4	-	-
Yes, in yard	43	10.8	-	-
Yes, nearby	66	16.6	-	-
No	172	43.2	-	-
**Do you have electricity in your home? (*n* = 399)**	-	-	-	-
Yes	332	83.2	-	-
No	67	16.8	-	-
**Which of the following best describes your home? (*n* = 398)**	-	-	-	-
Wood, zinc, mud	47	11.8	-	-
Brick or concrete	351	88.2	-	-
**Is anyone employed in your household? (it could be you) (*n* = 399)**	-	-	-	-
Yes	381	95.5	-	-
No	18	4.5	-	-
**Do you have water-borne sewage? (*n* = 397)**	-	-	-	-
Yes, inside house	83	20.9	-	-
Yes, in yard	35	8.8	-	-
Yes, nearby	19	4.8	-	-
No	260	65.5	-	-

Med, median.

Most participants (*n* = 317; 79.6%) had less than a secondary school diploma. Overall, 144 (36.3%) were self-employed in the informal sector, 130 (32.7%) in the formal sector, and only 30 (7.6%) were employed full-time. Most participants (381; 95.5%) reported at least one employed person in their household, 351 (88.2%) lived in brick or concrete block dwellings, and 332 (83.2%) reported having electricity.

However, 172 participants (43.2%) had no access to piped water, and 260 (65.5%) had no wastewater drainage pipe.

Most respondents (*n* = 324; 81.2%) rated their health status as good or excellent, and 52 (13.0%) reported a chronic condition.

Table 3 presents the results on affiliation. The median number of visits to the facility by respondents over the past 2 years was 6 (IQR: 4.0–10.0), and 79 respondents (19.8%) had been using the facility for 5 years or more.

**TABLE 3:** Affiliation of the respondents to the primary care facilities.

Designation	*n*	%
**All users’ affiliation (*n* = 399)**		
High affiliation	188	47.1
Moderate affiliation	160	40.1
Low affiliation	51	12.8
**Duration of use of the facility (*n* = 399)**		
Less than 6 months	49	12.3
Between 6 months and 1 year	89	22.3
1–2 years	98	24.6
3–4 years	76	19.0
5 years or more	79	19.8
Too variable to be precise	8	2.0
**Reason for choosing the health facility (*n* = 399)**		
Because it is close to where I live or work	214	53.6
Because I can pay the costs	118	29.6
Because it offers high-quality healthcare	282	70.7
Because I’m registered here and I’m not allowed to go anywhere else	0	0.0
Because my insurance covers me here	7	1.8
Because I was sent here by another facility	10	2.5

Affiliation was classified as high for 188 users (47.1%), moderate for 160 users (40.1%), and low for 51 users (12.8%). The two main reasons for using the selected health facility were geographical proximity (*n* = 214; 53.6%) and perceived good quality of care (*n* = 282; 70.7%).

Table 4 shows the scores for the core functions of primary care and the overall primary care score. Coordination – health system (score 2.3) was rated poor, while first-contact access (score 2.8), first-contact utilisation (score 3.3), comprehensiveness (score 2.6), continuity (score 2.9), and coordination – information system (score 3.0) were rated as average. Only person-centred care was rated good (score 3.5). The overall primary care score was rated as average (score 2.9).

**TABLE 4:** Score for the core functions of primary care.

Function	Med	Q1–Q3	Scoring ≥ 3		Interpretation
			*n*	%	
First-contact utilisation (*n* = 399)	3.3	3.0–3.7	302	75.7	Average
First-contact access (*n* = 399)	2.8	2.6–3.2	196	49.1	Average
Comprehensiveness (*n* = 399)	2.6	2.4–2.8	58	14.5	Average
Continuity (*n* = 399)	2.9	2.5–3.1	174	43.6	Average
Person-centred care (*n* = 399)	3.5	3.2–3.8	351	88.0	Good
Coordination – Information system (*n* = 399)	3.0	2.7–3.3	277	69.4	Average
Coordination – Health system (only for people referred; *n* = 114)	2.3	1.9–3.1	39	34.2	Poor
Primary care performance score (*n* = 399)	2.9	2.7–3.0	123	30.8	Average

Med, Median.

Bivariate analysis showed no statistically significant association between overall primary care score and gender, age, mother tongue, employment status, education level, presence of a chronic physical, mental or emotional health condition, electricity, housing type, household employment, wastewater drainage, commune and urbanisation status (*p* > 0.05). In contrast, self-reported health status was significantly associated with primary care score (*p* = 0.0006), with progressively lower score observed among participants reporting poorer health. Access to piped water showed a significant association with primary care score (*p* = 0.0292), with slightly higher median score among respondents with piped water available in the yard or inside the house than among those without piped water. The institutional status of the primary care facility (public or private) was also significantly associated with the primary care score (*p* < 0.001), with private facilities reporting slightly higher score (2.98) than the public facilities (2.84) (see Table 5).

**TABLE 5:** Association between the selected characteristics and the primary care score.

Variable	Med	Q1–Q3	*p*-value
**Age**	-	-	0.8328
**Gender**	-	-	0.9902
Male	2.88	2.66–3.00	-
Female	2.88	2.66–3.05	-
**Mother tongue**	-	-	0.76567
Fongbé	2.88	2.66–3.03	-
Adja	2.83	2.55–2.97	-
Aïzo	2.93	2.67–3.05	-
Toffin	2.91	2.74–3.13	-
Mina	2.95	2.71–3.18	-
Goun	2.84	2.68–2.94	-
Other	2.83	2.57–3.03	-
**Employment of patient**	-	-	0.2036
Full-time employee	2.90	2.72–3.05	-
Part-time employees	2.90	2.76–3.03	-
Self-employed (informal sector)	2.93	2.78–3.06	-
Self-employed (formal sector)	2.75	2.57–2.96	-
Student	2.84	2.59–2.98	-
Homemaker	2.84	2.72–3.07	-
Retired or pensioner	3.35	3.18–3.36	-
Unemployed	2.93	2.68–3.12	-
**Education level**	-	-	0.4356
Never went to school	2.80	2.65–3.01	-
Did not finish primary school	2.87	2.67–3.02	-
Completed primary school	2.88	2.7–2.97	-
Did not complete secondary school	2.91	2.7–3.05	-
Secondary school diploma	2.83	2.64–3.08	-
Higher education diploma, for example, bachelor’s degree, master’s degree	2.89	2.65–3.03	-
**Health status (Self-reported)**	-	-	**0.0006**
Excellent	2.93	2.67–3.05	-
Very good	2.92	2.75–3.09	-
Good	2.88	2.66–3	-
Fair	2.78	2.57–2.94	-
Poor	2.74	2.47–2.88	-
**Presence of a chronic physical, mental, or emotional health condition**	-	-	0.6915
Yes	2.88	2.65–3.07	-
No	2.88	2.67–3.03	-
**Piped water**	-	-	**0.0292**
Yes, inside house	2.90	2.65–3.07	-
Yes, in yard	2.93	2.78–3.07	-
Yes, nearby	2.89	2.77–3.07	-
No	2.83	2.6–2.98	-
**Electricity**	-	-	0.1053
Yes	2.89	2.67–3.05	-
No	2.78	2.6–3.01	-
**Housing type**	-	-	0.5948
Wood, zinc, mud	2.89	2.73–3.04	-
Brick or concrete	2.88	2.65–3.03	-
**Employment in household**	-	-	0.1789
Yes	2.88	2.67–3.05	-
No	2.72	2.5–2.95	-
**Wastewater drainage pipes**	-	-	0.7040
Yes, inside house	2.90	2.64–3.08	-
Yes, in yard	2.83	2.59–3.06	-
Yes, nearby	2.93	2.74–2.99	-
No	2.88	2.69–3.02	-
**Institutional status**	-	-	**< 0.001**
Private	2.98	2.81–3.13	-
Public	2.84	2.62–2.98	-
**Commune**	-	-	0.31156
Abomey-Calavi	2.88	2.65–3.03	-
So-Ava	2.88	2.72–3.13	-
**Urbanisation status**	-	-	0.1068
Rural	2.84	2.66–2.98	-
Urban	2.90	2.66–3.07	-

Note: Bold values indicate statistically significant differences (*p* < 0.05).

Med, median.

## Discussion

### Key findings

The findings highlight a young (median age of 28 years) and predominantly female (90.5%) study population. Most participants had less than a secondary school diploma and were mainly self-employed in the informal or formal sector. Despite relatively favourable housing conditions for most participants (brick or concrete dwellings and access to electricity), substantial proportions lacked access to piped water (43.2%) and wastewater drainage (65.5%). Nearly half of respondents (47.1%) reported high affiliation with their primary care facility, and 40.1% reported a moderate affiliation. The main reasons for choosing a facility were perceived quality of care (70.7%) and geographical proximity (53.6%). Only 19.8% had used the same facility for 5 years or more. The overall primary care performance score was rated as average (median 2.9), which is slightly below the threshold considered the minimum for acceptable quality of care.^17^ Person-centred care received a good rating (median 3.5), whereas coordination–health system (median 2.3) was rated poorly. Other core functions were rated as average. Primary care score did not differ significantly across most socio-demographic variables but showed statistically significant associations with respondents’ self-rated health (*p* = 0.0006), and access to piped water (*p* = 0.0292), as well as with the institutional status of the health facility (public or private) (*p* < 0.001).

### Comparison with existing literature

The comparison of the Abomey-Calavi – So-Ava results with PCAT studies conducted in South Africa, Kenya and Uganda^15,16,18^ shows broadly similar patterns but also some contextual differences. The overall primary care performance score in Abomey-Calavi – So-Ava was slightly lower than that reported in South Africa and Uganda but higher than that observed in Kenya. These differences may partly reflect variations in study settings. For instance, while the Abomey-Calavi – So-Ava health district has a mix of urban and rural settings, the Kenyan study was conducted in private-sector facilities in metropolitan areas, whereas the Ugandan study focused on rural public-sector facilities. Such contextual differences in health system organisation and population characteristics may influence patient perceptions of care quality. In Abomey-Calavi – So-Ava, the lowest score (2.3) was attributed to the function related to coordination within the health system, similar to findings from Uganda but lower than those reported in South Africa (3.3) and Kenya (2.9). This result suggests limited collaboration and communication between primary care providers (including general practitioners) and providers at secondary-level services, particularly the district hospital, as highlighted in a previous study on referral systems in Benin.^31^ Furthermore, in Benin, patients can access secondary and tertiary levels of care without a formal referral from primary care facilities. This open access to higher levels of care may reduce the incentive to follow referral pathways, thereby weakening the coordinating role of primary care, a pattern also observed in the Kenyan PCAT study.^32^ The second aspect of coordination (information system) received an average score (3), as in Uganda, Kenya, and South Africa.^15,16,30^ Although it still needs a lot of improvement, this average score can be linked to the habit of primary care providers in Benin of explaining the results of investigations when giving them to users. Moreover, it may be explained by the interprofessional collaboration between the providers working in the primary care facilities and those in the community, as found by Ashcroft et al.^33^ and Bouton et al.^34^ However, the lack of efficient electronic records can limit the availability and transfer of information to facilitate the development of an appropriate management plan.^17^ The person-centredness function received the highest score (3.5), a result similar to that reported in Uganda. In the context of Benin, however, this finding was somewhat surprising. The items used to assess this domain explored several aspects of the patient–provider relationship, including whether health workers greet patients appropriately, explain health problems and treatments clearly, allow patients to express their concerns, involve them in decision-making regarding their care, and their overall satisfaction with the care received. In contrast, one of the most frequently reported complaints from communities in Benin concerns the poor reception and interaction with health workers, including inappropriate or hurtful remarks.^35^ Given these recurrent concerns, the relatively high score observed for patient-centred care appears unexpected. Several explanations may explain this finding. Firstly, social desirability bias may have influenced participants’ responses. Because many of the questions relate directly to the behaviour and attitudes of health workers, patients may have been reluctant to provide negative assessments for fear of penalising them. Secondly, it is possible that what communities refer to as ‘poor reception’ extends beyond the dimensions captured by the PCAT items. For example, a recent qualitative study conducted in Benin showed that populations may also interpret poor reception as including poor hygiene conditions in health facilities or the high cost of care, which they sometimes attribute to the behaviour of certain health workers. These aspects are not directly captured by the PCAT patient-centredness items. Finally, it is also possible that the Abomey-Calavi – So-Ava health district performs better than other districts in the country in terms of patient reception and person-centred care. Additional studies conducted in other regions of Benin would help to assess better whether this finding reflects a broader national pattern or a context-specific situation. Regarding the first-contact function, users in this study rated first-contact access to primary care as average (median 2.8), a result comparable to those reported in Uganda (2.6), Malawi (2.3), and South Africa (2.5).^15,18,36^ Access to care is a well-documented challenge in sub-Saharan Africa. Financial constraints and geographical factors, including the distance between health facilities and users’ residences and transportation costs, are among the most frequently reported barriers to accessing primary care services.^37^ Stakeholders in the Abomey-Calavi – So-Ava district also highlighted financial barriers as a key challenge during the dissemination workshop of this study held in June 2025. In addition, the district includes communities living in lacustrine villages or in areas located far from the main roads, while most health facilities are situated along major road networks. These geographical constraints may partly explain the moderate performance observed for first-contact access in the district.

The two remaining functions, continuity of care and comprehensiveness, also received average scores (2.9 and 2.6, respectively), although these scores remain below the threshold considered acceptable for good-quality care. These findings are similar to those reported in Uganda.

However, the comprehensiveness score in Abomey-Calavi – So-Ava is higher than that observed in metropolitan private facilities in Kenya (2.1). This difference may be explained by the fact that some public sector–led interventions (such as vaccination programmes) are not always available in private facilities, or by differences in patients’ awareness of the services offered. The latter explanation may also apply to Benin, where patients tend to selectively use health facilities based on the services they perceive to be best provided there. Indeed, in many African settings, including Benin, primary care facilities – particularly public ones – are often perceived as best suited to maternal and child health services and the management of minor acute illnesses (such as malaria and common flu-like conditions). This perception may be rooted in the historical development of primary care in low- and middle-income countries, which has often been selective and driven by vertical, disease-oriented programmes.^10^ Another explanation for the relatively low comprehensiveness score may be the actual unavailability of certain services, as suggested by the participants’ feedback during the dissemination workshop. Moreover, the 2018 SARA in Benin showed that the service availability index in the Atlantique department (where the Abomey-Calavi – So-Ava district is located) was only around 30%, indicating substantial gaps in the range of services provided.^38^ Primary care performance in this study showed statistically significant associations with respondents’ self-rated health and access to piped water. The institutional status of the health facility (public or private) is also associated with primary care performance, with private facilities having a slightly higher score. The association between self-rated health and primary care performance is consistent with findings from other settings, including the Amathole District in South Africa, Korea, and Tibet (China), where better perceived health was associated with higher primary care score.^30,39,40^ This suggests a consistent relationship between patients’ perceived health status and their evaluation of primary care quality across different contexts. In contrast, no significant association was found with age, gender, education level, or chronic conditions. While the absence of associations with age, gender, and education aligns with findings from South Africa, the lack of association with chronic conditions differs from their results, in which patients with chronic illnesses reported lower score. Furthermore, the association between access to piped water and primary care performance may partly reflect the influence of living conditions observed in other studies, although the direction of association may vary.

### Implications

These findings highlight priority areas for strengthening primary care in Benin. Strengthening the coordination role of primary care should be prioritised through improved referral systems, better communication across levels of care, and enhanced use of health information systems. Improving access remains essential, requiring continued expansion of financial protection mechanisms such as *Assurance Maladie du projet Assurance pour le Renforcement du Capital Humain* (AM-ARCH), as well as targeted strategies to reach underserved and hard-to-access populations. Efforts should also focus on enhancing the comprehensiveness of services at the primary care level through investments in workforce capacity, equipment, and service integration. The good performance observed for person-centred care represents an asset that should be sustained through continued emphasis on patient engagement and respectful care.

In addition, this study highlights several areas for future research. Further analyses of PCAT data are needed to better understand the determinants of primary care performance in the Beninese context, particularly the roles of socioeconomic factors and facility-related factors. Advanced analytical approaches, such as dominance analysis, could provide more precise information on the relative contribution of different core functions of primary care to overall performance. The association between self-rated health and primary care performance warrants further investigation to better understand its direction and underlying mechanisms. Finally, this study should be conducted in other health districts in Benin and at the national level to assess the current situation nationwide.

### Strengths and limitations

To the best of our knowledge, this is the first study to assess the core functions of primary care in Benin. The study obtained the required sample size and followed the intended sampling method. The results can therefore be generalised to the health district. However, it might have been helpful to triangulate the patients’ scores with those of the managers or healthcare workers to get a more complete picture of the core functions. One selected healthcare facility declined participation immediately before data collection, resulting in the loss of six eligible participants. Consequently, the final sample included 399 participants instead of the planned 405. However, the achieved sample size remained above the minimum required sample size of 384.

## Conclusion

The overall primary care score was average in the Abomey-Calavi – So-Ava health district. Coordination – health system was poor and requires significant strengthening; this domain demands greater attention in reinforcement policies. First-contact access, first-contact utilisation, comprehensiveness, continuity, and coordination – information system were average and can be further strengthened. Only person-centred care was good. The new regional version of the PCAT used in this study provided novel information on the performance of primary care in the health district.
